# Survival outcomes of axillary de-escalation following neoadjuvant chemo-immunotherapy in clinically node-positive triple-negative breast cancer: a national cancer database study

**DOI:** 10.3389/fimmu.2026.1892648

**Published:** 2026-07-08

**Authors:** Zheng Li, Zheng Qu, Shengbin Pei, Keyao Xian, Hongzhe Zhang, Xue Yang, Luxiao Zhang, Jennifer Q. Zhang, Oluwadamilola M. Fayanju, Jing Wang, Yi Fang

**Affiliations:** 1 Department of Breast Surgical Oncology, National Cancer Center/National Clinical Research Center for Cancer/Cancer Hospital, Chinese Academy of Medical Sciences and Peking Union Medical College, Beijing, China; 2 Department of Biostatistics, Epidemiology, and Informatics, Perelman School of Medicine, University of Pennsylvania, Philadelphia, PA, United States; 3 Division of Breast Surgery, Department of Surgery, Perelman School of Medicine, University of Pennsylvania, Philadelphia, PA, United States; 4 Rena Rowan Breast Center, Abramson Cancer Center, University of Pennsylvania, Philadelphia, PA, United States; 5 Penn Center for Cancer Care Innovation, Abramson Cancer Center, Philadelphia, PA, United States; 6 Leonard Davis Institute of Health Economics, University of Pennsylvania, Philadelphia, PA, United States

**Keywords:** axillary de-escalation, breast cancer, immunotherapy, lymph node metastasis, neoadjuvant chemotherapy

## Abstract

**Background:**

Adding immune checkpoint inhibitors (ICIs) to neoadjuvant chemotherapy (NAC) enhances systemic efficacy in triple-negative breast cancer (TNBC). However, its impact on the survival outcomes of axillary surgical de-escalation remains undefined. This study evaluates whether chemo-immunotherapy mitigates survival risks historically associated with omitting axillary lymph node dissection (ALND) for sentinel lymph node biopsy (SLNB) in clinically node-positive (cN+) disease.

**Methods:**

In this retrospective cohort study using the National Cancer Database (2018-2022), we identified women with cN1-3, cM0 TNBC who received NAC ± ICIs and achieved ypN0 (axillary pathological complete response), followed by axillary surgery (SLNB or ALND). Overall survival (OS) was evaluated using restricted mean survival time (RMST) and propensity score matching. Multivariable Cox models assessed the independent effect of surgical extent (SLNB vs. ALND) on OS.

**Results:**

Out of 1,315,170 breast cancer cases, 4,336 eligible patients were included. The therapeutic regimen significantly interacted with the survival impact of axillary de-escalation. With NAC alone, SLNB was independently associated with inferior OS compared to ALND [5-year OS: 83.0% vs. 87.8%, P = .027; adjusted hazard ratio (aHR)=1.63, 95% CI = 1.12-2.38, P = .01]. Strikingly, adding ICIs neutralized this disparity: in the NAC+ICI cohort, SLNB yielded OS comparable to ALND (5-year OS: 90.1% vs. 93.6%, P = .99; identical 48-month RMST), with no significant survival detriment in multivariable analysis (aHR=1.19, 95% CI = 0.54-2.61, P = .67).

**Conclusions:**

The enhanced systemic control conferred by ICIs appears to compensate for the reduced surgical clearance of the axilla when ALND is omitted, effectively mitigating the survival risks associated with omitting ALND in cN+ TNBC. These real-world findings suggest modern chemo-immunotherapy facilitates axillary de-escalation without compromising overall survival, establishing a compelling rationale for prospective clinical trials.

## Introduction

Triple-negative breast cancer (TNBC) accounts for approximately 15% to 20% of all breast cancer diagnoses and is characterized by an aggressive clinical phenotype, higher rates of metastasis, and poorer survival outcomes compared to other molecular subtypes (1). Historically, the cornerstone of management for early-stage TNBC has been neoadjuvant chemotherapy (NAC), which serves the dual purpose of downstaging the primary tumor and axilla to facilitate surgical resection while providing *in vivo* sensitivity testing of the tumor to systemic agents (2). In recent years, the treatment landscape for TNBC has undergone a paradigm shift with the integration of immunotherapy. Landmark trials, most notably the KEYNOTE-522 study, have demonstrated that the addition of immune checkpoint inhibitors (ICIs), such as pembrolizumab, to standard platinum-containing NAC significantly improves pathological complete response (pCR) rates and event-free survival (3, 4).

While systemic therapies have intensified, locoregional management has simultaneously trended toward de-escalation to minimize morbidity. For patients presenting with clinically node-positive (cLN+) disease, axillary lymph node dissection (ALND) was traditionally the standard of care to ensure local control and accurate staging. However, ALND is associated with significant long-term sequelae, including lymphedema, sensory neuropathy, and restricted shoulder mobility, which substantially diminish quality of life (5). Consequently, pivotal trials such as ACOSOG Z1071 and SENTINA have investigated the feasibility of de-escalating surgery to sentinel lymph node biopsy (SLNB) in patients who convert from cN1 to ycN0 following NAC (6, 7). These studies established that SLNB is technically feasible, yet concerns regarding false-negative rates and the potential for leaving residual nodal disease have led to cautious adoption, often requiring targeted axillary dissection (TAD) or dual-tracer mapping to ensure oncologic safety (8).

Despite these advances, a critical knowledge gap remains at the intersection of intensified systemic immunotherapy and de-escalated axillary surgery. Although ICIs have been shown to enhance pCR rates in the breast and lymph nodes, it remains unclear whether this improved systemic control translates into a survival benefit that allows for safer surgical de-escalation in node-positive patients (9). Specifically, in the era of immunotherapy, the robust systemic response induced by ICIs may mitigate the survival risks historically associated with omitting ALND in cLN+ patients. The randomized trials establishing the efficacy of ICIs were primarily powered to assess systemic endpoints (pCR and EFS) rather than to compare the specific survival impact of surgical approaches (SLNB vs. ALND) within the ICI-treated population (10). Therefore, real-world evidence is urgently needed to determine if the addition of ICIs renders the extent of axillary surgery less critical for overall survival, potentially sparing more women from the morbidity of full dissection.

To address this question, we utilized the National Cancer Database, a comprehensive clinical oncology database capturing approximately 70% of all newly diagnosed cancer cases in the United States (11). This study aimed to investigate whether the addition of ICIs to NAC facilitates the safe de-escalation of axillary surgery from ALND to SLNB in patients presenting with cN1–3 TNBC. By leveraging a large, contemporary cohort and utilizing propensity score matching and restricted mean survival time (RMST) analysis, we sought to isolate the interaction between systemic therapy type (NAC vs. NAC+ICIs) and surgical extent on overall survival. To our knowledge, this is one of the first large-scale analyses to specifically evaluate whether the survival equivalence of SLNB and ALND is dependent on the receipt of immunotherapy. The findings from this study have the potential to refine surgical guidelines for TNBC, supporting a more personalized, de-escalated approach that aligns surgical extent with the efficacy of modern systemic therapy.

## Methods

### Study design and data sources

As a collaborative effort between the American College of Surgeons and the American Cancer Society, the National Cancer Database (NCDB) is a comprehensive, hospital-based registry encompassing data on roughly 70% of malignant cancers diagnosed nationwide. It provides extensive information regarding patient demographics, comorbidities, tumor specifics, and overall survival (OS). Furthermore, the NCDB details the primary therapeutic modalities administered, such as surgery, radiotherapy, chemotherapy, immunotherapy, hormone therapy, and targeted therapy. The application, download, use, and analysis of PUF files in the study were all subject to the committee’s oversight, ensuring strict adherence to all established protocols.

**Figure 1:**
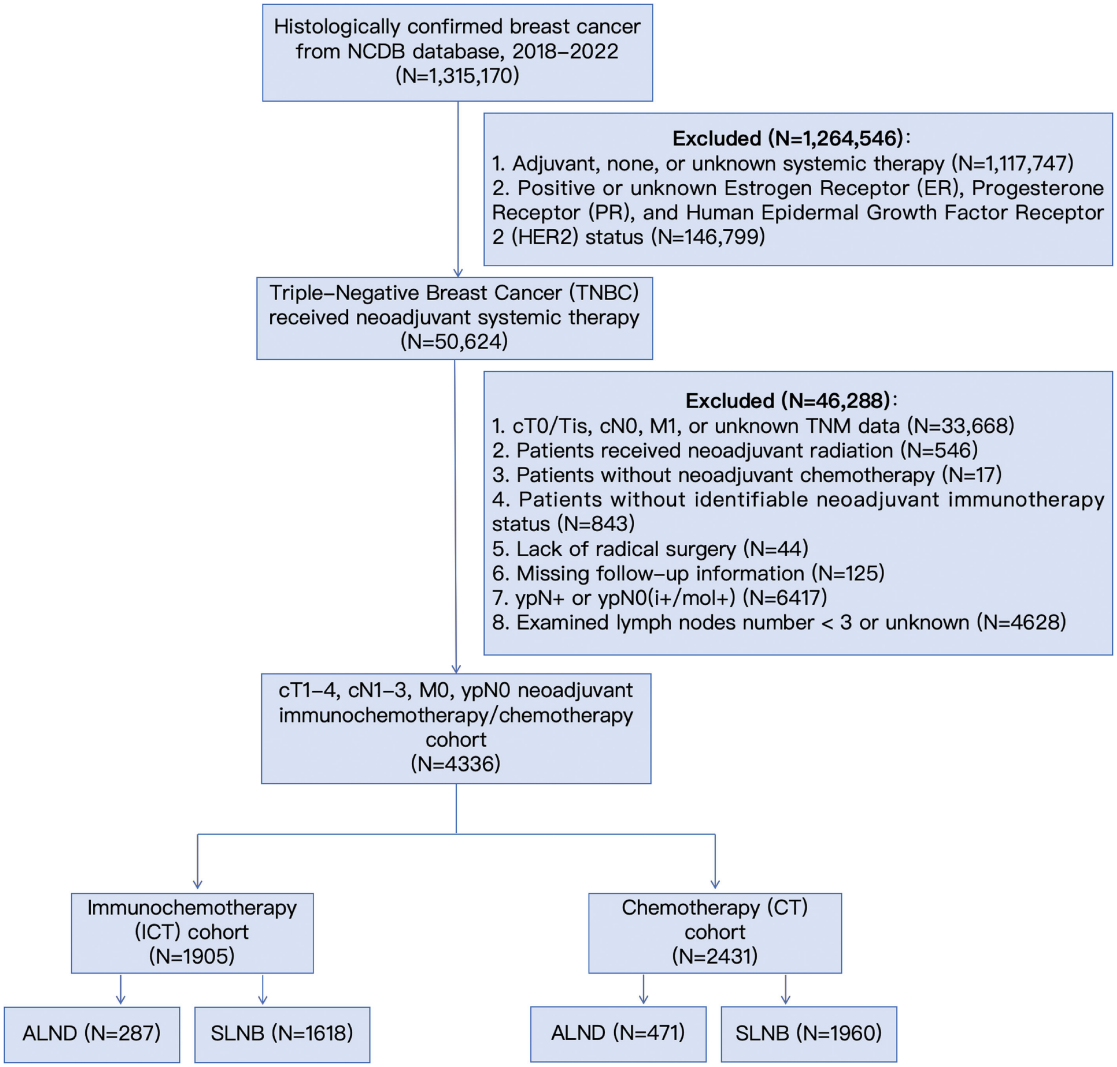
Flow diagram showing the inclusion and exclusion of patients in the study. NAC, neoadjuvant chemotherapy; ICIs, immune checkpoint inhibitors; SLNB, sentinel lymph node biopsy; ALND, axillary lymph node dissection.

The updated database was queried for patients with histologically confirmed breast cancer diagnosed in immunotherapy era, 2018-2022. Complete datasets were identified for TNBC treated with neoadjuvant systemic therapy. NAC in the study was defined by an interval from chemotherapy initiation to surgery of ≥84 and ≤270 days (12). It is established that in the cohort of clinically node-positive (cLN+) breast cancer patients who achieve a pathologically negative nodal status following NAC, a low incidence of nodal recurrence is observed when a minimum of three negative sentinel lymph nodes are identified during SLNB. This finding justifies the omission of routine ALND in this subgroup (13). Considering this evidence, the present study selectively enrolled cases with three or more harvested sentinel nodes. Patients were excluded according to the following criteria: (i) with cT0/Tis (occult or *in situ* disease), cN0 (clinically node-negative disease), M1 (distant metastases), ypN+ (pathologically node-positive disease after neoadjuvant therapy), ypN0(i+/mol+) (isolated tumor cells or micrometastases detected by immunohistochemistry or molecular methods), or unknown TNM staging information; (ii) with preoperative radiotherapy; (iii) with unclear records of NAC or immunotherapy status; (iv) undergone surgery other than mastectomy or breast-conserving surgery (BCS); (v) with <3 or unknown number of examined lymph nodes. Finally, NAC cohort and NAC plus ICIs cohort were identified, consisting of patients with cT1-4, cN1-3, M0 disease (clinically node-positive, non-metastatic tumors) who achieved ypN0 status (pathologically node-negative after neoadjuvant therapy, confirmed by either SLNB or ALND showing no residual tumor deposits), as shown in **Figure 1**. Axillary surgery was categorized according to the NCDB data dictionary (variable: RX_SUMM_SCOPE_REG_LN_2012) into four groups: no surgery (code 0), SLNB alone (code 2), ALND alone (codes 3-5), and SLNB followed by ALND (codes 6-7). Axillary surgery was reclassified into two groups: SLNB alone, and ALND (encompassing both ALND alone and SLNB with completion ALND). Since TAD lacks a specific code in the NCDB, it is assumed to be categorized as SLNB alone, or as part of the ALND group if a completion dissection was performed.

### Statistical analysis

The outcomes of interest were overall survival. Patient characteristics were summarized as number (%) for categorical variables and as median (interquartile range [IQR]) for continuous variables. Differences were tested with chi-square for categorical variables and with analysis of variance (ANOVA) for continuous variables. Unadjusted overall survival was estimated with the Kaplan-Meier method, and log-rank tests were used to compare survival between groups. To minimize selection bias, a propensity score matching (PSM) analysis was conducted. Patients were first stratified into two cohorts based on their treatment regimen: NAC alone or NAC combined with ICIs. Within each of these two cohorts, a separate PSM was performed to balance the baseline characteristics between patients undergoing ALND and SLNB. The propensity scores were calculated based on a comprehensive set of clinical, pathological, demographic, and socioeconomic covariates, including age, race, facility type, year of diagnosis, ypT, cN, cT, grade, Charlson-Deyo score, urban/rural location, education level, and receipt of postoperative radiation or chemotherapy. A 1:1 nearest neighbor matching algorithm was employed with a caliper width of 0.02. The PSM process was shown in **Supplementary Figure 1**.

The Kaplan-Meier analysis was limited by unequal follow-up durations between groups, especially after 48 months, potentially affecting the reliability of the results. Consequently, we employed RMST analysis to compare survival over an identical time horizon, ensuring a more robust assessment. Subsequently, a multivariable regression model was developed to isolate the effect of axillary surgery on prognosis. The model incorporated axillary surgery type (ALND or SLNB) along with a comprehensive set of covariates, including critical clinicopathological characteristics and radiotherapy status, to ensure a thorough adjustment for potential confounders. Hazard ratios (HRs) and 95% confidence intervals (CIs) were reported. Significance levels were defined as two-sided *p* <.05. All statistical analyses were conducted using R software (version 4.4.1), GraphPad Prism (version 8.0.1) and IBM SPSS software (version 25.0). Data analysis was conducted from December 2025 to February 2026. This project was deemed exempt from institutional review board (IRB) review because it used only de-identified, publicly available data from the NCDB in accordance with institutional policies.

## Results

### Cohort information

The study included 4336 patients with cN1-3, cM0 TNBC who received NAC ± ICIs and achieved ypN0 (axillary pathological complete response). Patient were further subclassified by those who had SLNB alone and those who had ALND to compare survival outcome, including 2431 patients in the NAC cohort (ALND: n=471; SLNB: n=1960) and 1905 patients in the NAC+ICIs cohort (ALND: n=287; SLNB: n=1618), as shown in **Figure 1**; **Table 1**. The mean age at diagnosis was 52.4 years [standard deviation (SD) 13.0]. Most of patients had a Charlson-Deyo comorbidity score of 0 [n=3694 (85.2%)]. Annual number of patients increased from 591 (13.6%) in 2018 to 1236 (28.5%) in 2022. The majority of patients were White [n=2873 (66.3%)]. Patients were predominantly treated at academic centers [n=1295 (29.9%)] and covered by Private Insurance/Managed Care [n=2671 (61.6%)]. SLNB was the most frequently performed axillary procedure [n=3578 (82.5%)]. The predominant clinical T stage was cT2 [n=2487 (57.4%)], while post-neoadjuvant pathological assessment revealed that most patients achieved ypT0/Tis status [n=3112 (71.8%)]. Regarding tumor grade, most cases were poorly differentiated [n=3768 (86.9%)]. 3689 (85.1%) patients resided in metropolitan areas. Analysis of neighborhood-level socioeconomic indicators showed that the largest group of patients lived in areas with 5.0%-9.0% of residents without a high school diploma [n=1042 (24.0%)] and in regions with a median household income above $74,063 [n=1368 (31.5%)]. Neoadjuvant immunotherapy, postoperative radiotherapy, and postoperative adjuvant chemotherapy were administered to 1905 (43.9%), 3523 (81.3%), and 1430 (33.0%) of the patients, respectively.

**Table 1:** Baseline co-variates of patients diagnosed with TNBC.

Characteristics	Overall (N = 4336)
Age	
Mean (SD)	52.4 (13.0)
Median [IQR]	52.0 [42.0, 62.0]
Age category (%)	
<40	819 (18.9)
40-49	1014 (23.4)
50-59	1177 (27.1)
≥60	1326 (30.6)
Year of diagnosis (%)	
2018	591 (13.6)
2019	742 (17.1)
2020	743 (17.1)
2021	1024 (23.6)
2022	1236 (28.5)
Laterality (%)	
left	2218 (51.2)
right	2115 (48.8)
Unknown	3 (0.1)
Race (%)	
White	2873 (66.3)
Black or African American	1117 (25.8)
Asian and Pacific Islander	220 (5.1)
American Indian or Alaska Native	20 (0.5)
Unknown	106 (2.4)
Facility type (%)	
Academic/Research Program	1295 (29.9)
Community Cancer Program	199 (4.6)
Comprehensive Community Cancer Program	1176 (27.1)
Integrated Network Cancer Program	847 (19.5)
Unknown	819 (18.9)
Insurance (%)	
Government	1505 (34.7)
Private Insurance/Managed Care	2671 (61.6)
Not Insured	120 (2.8)
Unknown	40 (0.9)
Zip-code education (%)	
< 5.0% without high school diploma	795 (18.3)
5.0% - 9.0% without high school diploma	1042 (24.0)
9.1% - 15.2% without high school diploma	1014 (23.4)
>15.3% without high school diploma	732 (16.9)
Unknown	753 (17.4)
Zip-code income (%)	
< $46,277	539 (12.4)
$46,277 - $57,856	751 (17.3)
$57,857 - $74,062	920 (21.2)
>$74,063	1368 (31.5)
Unknown	758 (17.5)
Urban/rural location (%)	
Metro	3689 (85.1)
Rural	105 (2.4)
Urban	365 (8.4)
Unknown	177 (4.1)
Charlson-Deyo comorbidity index (%)	
0	3694 (85.2)
1	467 (10.8)
2	120 (2.8)
≥3	55 (1.3)
cT stage (%)	
cT1	884 (20.4)
cT2	2487 (57.4)
cT3	775 (17.9)
cT4	190 (4.4)
cN stage (%)	
cN1	3658 (84.4)
cN2	321 (7.4)
cN3	357 (8.2)
ypT stage (%)	
ypT0/ypTis	3112 (71.8)
ypT1	970 (22.4)
ypT2	213 (4.9)
ypT3	35 (0.8)
ypT4	6 (0.1)
Grade (%)	
Well differentiated	11 (0.3)
Moderately differentiated	455 (10.5)
Poorly differentiated	3768 (86.9)
Undifferentiated and anaplastic	2 (0.0)
Unknown	100 (2.3)
pCR (%)	
No	1224 (28.2)
Yes	3112 (71.8)
Lymphovascular Invasion (%)	
No	2289 (52.8)
Yes	441 (10.2)
Unknown	1606 (37.0)
Surgical margins (%)	
Negative	4198 (96.8)
Positive	58 (1.3)
Unknown	80 (1.8)
Surgery type (%)	
Breast-Conserving Surgery	2160 (49.8)
Total mastectomy	2176 (50.2)
Axillary surgery (%)	
ALND	758 (17.5)
SLNB	3578 (82.5)
Number of examined lymph nodes	
Median [Min, Max]	4.00 [3.00, 39.0]
Postoperative radiation (%)	
No	813 (18.8)
Yes	3523 (81.3)
Neoadjuvant immunochemotherapy (%)	
No	2431 (56.1)
Yes	1905 (43.9)
Postoperative chemotherapy (%)	
No	2906 (67.0)
Yes	1430 (33.0)

SLNB, sentinel lymph node biopsy. ALND, axillary lymph node dissection. cT stage, clinical T stage. cN stage, clinical N stage. ypT stage, post-therapy pathologic T stage; pCR, pathologic complete response.

### Axillary surgical management in the NAC and NAC plus ICIs cohorts

Patients in the NAC cohort (n= 2431) were stratified by surgical procedure into an ALND group [n=471 [19.4%)] and an SLNB group [n=1960 [80.6%)]. An initial comparison of baseline characteristics between the two surgical groups revealed significant differences in year of diagnosis (p < 0.001), surgery type (p = 0.045), clinical T stage (p = 0.004), and tumor grade (p = 0.027). To address these imbalances and mitigate potential selection bias, we performed a 1:1 propensity score matching as detailed in the Methods section. This procedure resulted in a well-balanced cohort of 898 patients, comprising 449 who underwent ALND and 449 who underwent SLNB (**Supplementary Table 1**). Among the 1905 patients in the NAC plus ICIs cohort, 287 underwent ALND and 1618 underwent SLNB. Significant differences were observed between the 2 groups regarding race (P < .01), surgery type (P < .001), and postoperative radiation (P < .01). After propensity score matching, the analysis included 550 patients, with 275 in the ALND group and 275 in the SLNB group, as shown in **Supplementary Table 2**.

**Figure 2:**
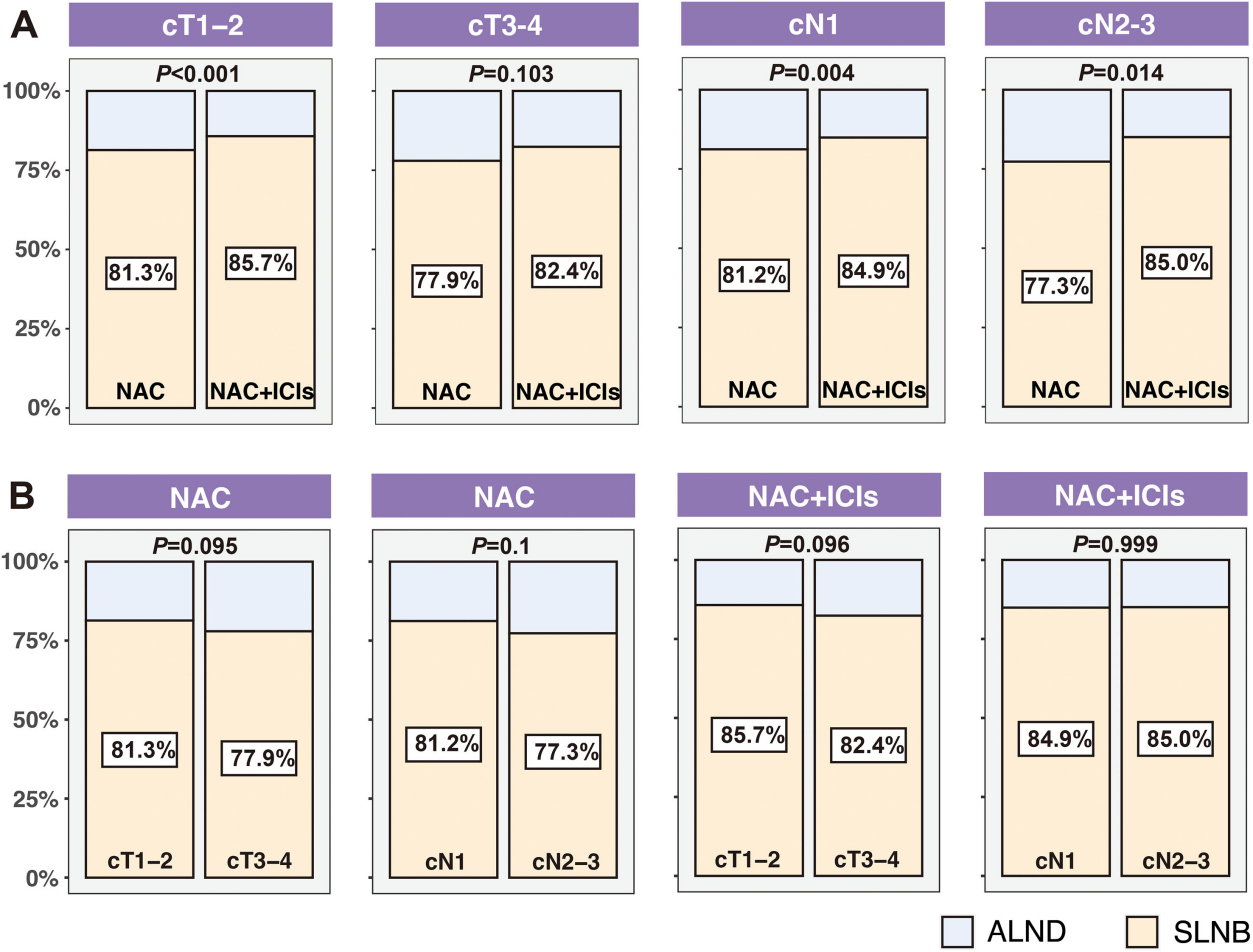
Patterns of axillary surgical management by treatment group in breast cancer patients. **(A)** Axillary surgical management patterns by clinical stage (cT1–2, cT3–4, cN1, and cN2–3). **(B)** Axillary surgical management patterns by neoadjuvant treatment regimen (chemotherapy alone vs. chemotherapy plus ICIs).

Stratification by clinical stage revealed that patients before PSM receiving NAC plus ICIs had higher rates of SLNB compared with the NAC group. This difference was significant for patients with cT1–2 disease (85.7% vs 81.3%; P < .01) but not for those with cT3–4 disease (82.4% vs 77.9%; P = .10). In terms of nodal involvement, the addition of ICIs was associated with increased SLNB use in both the cN1 (84.9% vs 81.2%; P < .01) and cN2-3 (85.0% vs 77.3%; P = .01) subgroups (**Figure 2**). Within the NAC cohort, axillary surgical strategy did not vary significantly by tumor stage (cT3–4 vs cT1-2: 77.9% vs 81.3%; P = .095) or nodal status (cN2–3 vs cN1: 77.3% vs 81.2%; P = .10). These findings were mirrored in the NAC plus ICIs cohort, where no significant differences were found between T stages (82.4% vs 85.7%; P = .096) or N stages (85.0% vs 84.9%; P > .99) as shown in **Figure 2**.

### Association of immunotherapy and axillary management with overall survival

To evaluate the survival impact of adding ICIs to NAC, patients were stratified by axillary surgical procedure (SLNB vs ALND), serving as a proxy for nodal disease burden. Among patients who underwent SLNB, the addition of ICIs was associated with significantly improved overall survival (5-year survival rate, 92.1% vs 88.7%; P = .029). Conversely, in the ALND cohort, adding ICIs did not yield a statistically significant survival benefit (5-year survival rate, 93.5% vs 86.7%; P = .59) (**Supplementary Figure 2**). Although the 5-year point estimates in the ALND group appeared numerically disparate, inspection of the data revealed that follow-up for the NAC plus ICIs group was largely censored by 48 months, rendering the 5-year estimate unstable. To address this, a RMST analysis was performed to ensure a robust comparison, as shown in **Supplementary Table 3**. The RMST analysis confirmed the lack of benefit in the ALND cohort [months (95%CI): 46.35 (45.5-47.1) vs 46.29 (45.7-46.8); P = .91] but corroborated the significant survival advantage in the SLNB cohort [months (95%CI): 46.58 (46.2-46.9) vs 46.07 (45.8-46.4); P = .03] (**Supplementary Figure 3**).

**Figure 3:**
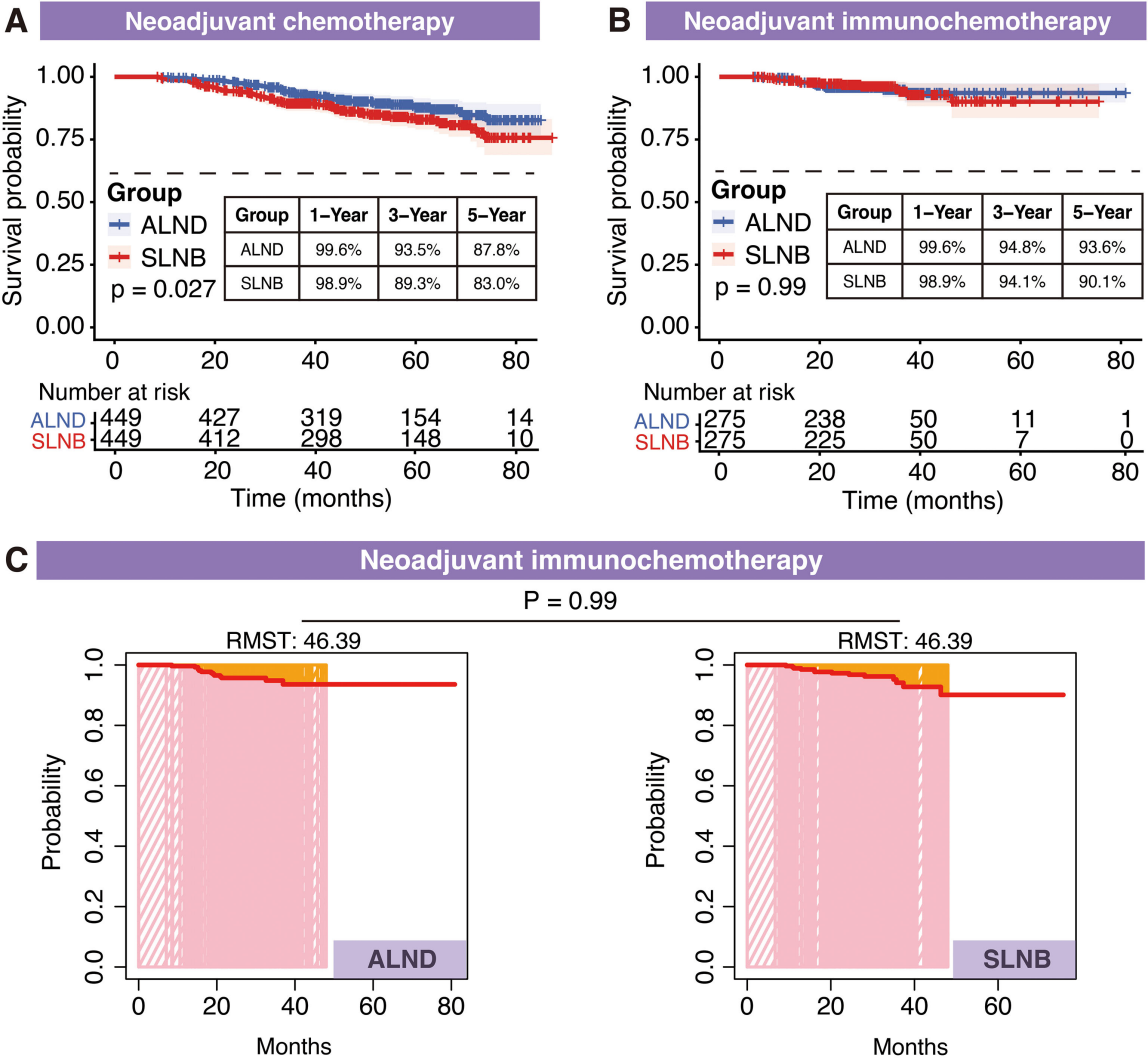
Survival analysis based on matched data stratified by ALND and SLNB in **(A)** neoadjuvant chemotherapy and **(B)** neoadjuvant immunochemotherapy cohort. **(C)** RMST analysis in neoadjuvant immunochemotherapy cohort of patients treated with ALND and SLNB. The truncation time was defined as 48 months; RMST, restricted mean survival time.

We further investigated the association between axillary surgical procedure and survival within the PSM cohorts. Among patients treated with NAC alone, ALND was associated with a significant survival benefit compared with SLNB (3-year survival rate, 93.5% vs 89.3%; 5-year survival rate, 87.8% vs 83.0%; P = .027). In contrast, among patients treated with NAC plus ICIs, no significant survival difference was observed between the ALND and SLNB groups (3-year survival rate, 94.8% vs 94.1%; 5-year survival rate, 93.6% vs 90.1%; P = .99), as shown in **Figure 3**. Because follow-up in the immunotherapy cohort was largely censored at 48 months, RMST analysis was performed to validate these findings (**Supplementary Table 4**). The analysis confirmed the lack of difference in the NAC plus ICIs cohort, yielding identical values of 46.39 months for both the ALND [months (95%CI): 46.39 (45.5-47.2)] and SLNB [months (95%CI): 46.39 (45.5-47.3)] groups (P > .99).

### Multivariable-adjusted analysis

To account for the complexity of survival determinants and potential interactions, a multivariable analysis was performed using the propensity score–matched data, adjusting for key clinicopathological covariates. In the NAC cohort, independent factors associated with decreased survival included older age (hazard ratio [HR], 1.03; 95% CI, 1.01-1.04; P < .01), higher Charlson-Deyo comorbidity score (HR, 2.96; 95% CI, 1.24-7.05; P = .01), and receipt of SLNB (HR, 1.63; 95% CI, 1.12-2.38; P = .01). Additionally, advanced T stage was significantly associated with mortality risk [T1: HR, 1.78 (95% CI, 1.17-2.72), P = .01; T2: HR, 3.18 (95% CI, 1.78-5.68), P < .01; and T3: HR, 9.73 (95% CI, 4.28-22.12), P < .01].

In the multivariable analysis of the NAC plus ICIs cohort, independent predictors of decreased survival included older age [adjusted hazard ratio (aHR), 1.03; 95% CI, 1.00-1.06; P = .03] and advanced tumor stage [T2: aHR, 4.70 (95% CI, 1.31-16.94), P = .02; T3: aHR, 27.80 (95% CI, 2.98-259.58), P < .01]. Notably, unlike in the NAC-only cohort, axillary surgical procedure (SLNB vs ALND) was not a significant prognostic factor in this group (aHR, 1.19; 95% CI, 0.54-2.61; P = .67), as shown in **Table 2**.

**Table 2:** Multivariable survival analysis stratified by clinicopathological factors in NAC cohort and NAC+ICIs cohort.

Characteristics	NAC cohort		NAC+ICIs cohort	
	Adjusted HR (95%CI)	P	Adjusted HR (95%CI)	P
Age*	1.03 (1.01 - 1.04)	<0.01	1.03 (1 - 1.06)	0.03
Charlson-Deyo score				
0	1 (Ref)		1 (Ref)	
1	1.4 (0.83 - 2.36)	0.21	0.76 (0.18 - 3.29)	0.72
2	2 (0.72 - 5.56)	0.18	NA	NA
>=3	2.96 (1.24 - 7.05)	0.01	3.34 (0.42 - 26.46)	0.25
Radiation				
No	1 (Ref)		1 (Ref)	
Yes	0.83 (0.53 - 1.3)	0.41	0.51 (0.22 - 1.17)	0.11
Axillary treatment				
ALND	1 (Ref)		1 (Ref)	
SLNB	1.63 (1.12 - 2.38)	0.01	1.19 (0.54 - 2.61)	0.67
ypT stage †				
T0	1 (Ref)		1 (Ref)	
T1	1.78 (1.17 - 2.72)	0.01	1.17 (0.39 - 3.52)	0.78
T2	3.18 (1.78 - 5.68)	<0.01	4.7 (1.31 - 16.94)	0.02
T3	9.73 (4.28 - 22.12)	<0.01	27.8 (2.98 - 259.58)	<0.01
Grade †				
Moderately differentiated	1 (Ref)		1 (Ref)	
Poorly differentiated	1.62 (0.86 - 3.04)	0.13	0.61 (0.18 - 2.13)	0.44

Cl, confidence interval. ALND, axillary lymph node dissection. SLNB, sentinel lymph node biopsy. NAC, neoadjuvant chemotherapy; ICIs, immune checkpoint inhibitors. NA=not applicable. Ref, reference.

*Per 1-year increase.

†T4, well/unknown differentiated was excluded due to the small sample size.

## Discussions

In this analysis of patients with clinically node-positive (cLN+) TNBC who achieved ypN0 status (pathologically node-negative after neoadjuvant therapy, i.e., no residual tumor deposits in lymph nodes), we observed a significant interaction between the systemic therapeutic regimen and the oncologic safety of axillary surgical de-escalation. The primary finding of this study is that the addition of ICIs to neoadjuvant chemotherapy attenuated the survival disparity historically observed between SLNB and ALND. Specifically, among patients receiving chemo-immunotherapy, those undergoing SLNB experienced overall survival outcomes comparable to those undergoing ALND. In contrast, within the cohort treated with NAC alone, axillary de-escalation to SLNB was independently associated with inferior survival compared with ALND. These data suggest that the robust systemic and locoregional control provided by ICIs may compensate for the reduced extent of surgical nodal clearance associated with SLNB.

These findings represent a logical progression in the decades-long evolution of breast cancer surgery, shifting from the Halstedian paradigm of radical resection to the Fisherian hypothesis that breast cancer is a systemic disease (14). Just as the NSABP B-04 trial demonstrated that radical mastectomy offered no survival benefit over simple mastectomy when systemic disease was the primary driver of mortality (15), our results suggest that in the era of potent immunotherapy, the extent of axillary surgery may be safely minimized. The equivalence of SLNB and ALND observed exclusively in the immunotherapy cohort suggests that ICIs may effectively eliminate residual tumor deposits in regional lymph nodes, reducing the risk of future recurrence. The strong association between high level of tumor-infiltrating lymphocytes (TILs) abundance and improved survival in TNBC reflects the profound impact of immune activation and intercellular dynamics, supporting the rationale for integrating immunotherapy into treatment paradigms (16). It is biologically plausible that the sustained immune activation induced by adjuvant ICIs eradicates microscopic deposits in non-sentinel nodes that might otherwise be missed by SLNB (17), thereby neutralizing the risk of regional recurrence.

The safety of omitting ALND should also be interpreted in the context of adjuvant radiotherapy, which serves as a critical modality for locoregional control. Although the AMAROS trial was restricted to patients with clinically node-negative (cLN-) disease, without neoadjuvant therapy, which limits its direct applicability to our cohort of patients who were clinically node-positive before NAC and achieved node negativity afterward. Nevertheless, its findings regarding the role of radiotherapy in locoregional control are relevant: The trial established that axillary radiotherapy provides comparable regional control to ALND in cN0 patients with positive sentinel nodes but with significantly lower rates of lymphedema (18). In our study, the majority of patients received adjuvant radiation. It is likely that the combination of systemic immunotherapy and regional nodal irradiation provides a synergistic effect, creating a “therapeutic field” that effectively controls residual axillary disease without the morbidity of dissection. Crucially, the proportion of patients receiving radiotherapy was well-balanced between the NAC and NAC+ICIs cohorts, which further strengthens the robustness of our findings. Meanwhile, consistent with findings from the NSABP B-51/RTOG 1304 trial, which showed no survival benefit of regional nodal irradiation (RNI) for patients achieving axillary pathological complete response (cLN+ to ypN0) (19), our study observed that radiotherapy was not associated with improved prognosis. Multivariate analysis confirmed this lack of significance in both the NAC and NAC+ICIs treatment arms. Therefore, variations in radiotherapy use are unlikely to be a significant confounding factor influencing our findings.

A notable and complex finding of this investigation is the inferior survival associated with SLNB in the NAC-alone cohort. This finding should be interpreted cautiously, recognizing both biological mechanisms and potential selection biases inherent to observational data. Biologically, without ICIs, there is no additional systemic treatment to address residual nodal disease. Without the enhanced surveillance provided by immunotherapy, microscopic deposits missed by SLNB may possess a higher potential for progression to distant metastasis, making the thorough clearance of ALND therapeutically relevant in this subgroup (13). However, this survival difference may also reflect unmeasured confounding by indication. In contemporary practice, patients who do not receive ICIs often represent a demographic with specific contraindications, such as autoimmune disorders, or those with frailty and significant comorbidities that preclude the use of checkpoint inhibitors (20). It is plausible that the decision to perform SLNB rather than ALND in this specific subset was influenced by clinical judgment regarding patient vulnerability. Surgeons may have favored less invasive procedures for patients deemed poor candidates for extensive dissection. Although propensity score matching balanced observable characteristics, administrative databases cannot fully capture the granular performance status that drives such clinical decision-making.

While this study utilized ypN0 as the primary selection criterion for de-escalation, future strategies will likely require more precise biomarkers to identify ideal candidates for limited surgery. Emerging technologies such as circulating tumor DNA (ctDNA) analysis have shown promise in detecting minimal residual disease and predicting relapse in TNBC (21). Integrating ctDNA status with clinical response to chemo-immunotherapy could refine surgical decision-making, allowing surgeons to distinguish patients who have achieved true biological clearance from those who harbor occult resistant disease requiring aggressive local therapy. Furthermore, the evaluation of residual cancer burden (RCB) index and stromal tumor-infiltrating lymphocytes may provide additional granularity to guide post-neoadjuvant locoregional management (22).

### Limitations

The interpretation of these results should be considered within the context of several limitations inherent to retrospective registry analyses (11). First, the National Cancer Database (NCDB) does not capture locoregional recurrence events; therefore, overall survival was utilized as the primary surrogate for oncologic safety. While survival is the definitive endpoint, it is influenced by post-recurrence therapies, and future studies with recurrence data are needed to confirm these findings. Second, detailed information regarding the SLNB technique, such as the use of dual tracers or the retrieval of clipped nodes, was unavailable. Variations in surgical quality could theoretically influence outcomes and false-negative rates. Additionally, the NCDB does not distinguish between standard SLNB and targeted axillary dissection (TAD), which combines removal of the clipped node with sentinel node dissection; patients undergoing TAD without completion ALND are coded as SLNB alone. This is a relevant limitation, as ACOSOG Z1071 and other studies have demonstrated that TAD with retrieval of 2 or more nodes achieves a low false-negative rate comparable to ALND in selected patients, and this technique may be increasingly adopted in contemporary practice. Third, although we adjusted for comorbidity scores, residual confounding regarding the omission of immunotherapy or ALND cannot be entirely excluded. Finally, the follow-up period for the immunotherapy cohort reflects the recent approval of these agents, and continued monitoring is essential to ensure that the survival equivalence persists over the long term.

## Conclusions

In conclusion, the integration of immune checkpoint inhibitors into neoadjuvant therapy for clinically node-positive TNBC appears to facilitate the safe de-escalation of axillary surgery. While ALND was associated with a survival advantage in patients treated with chemotherapy alone, likely attributable to a complex interplay of therapeutic necessity and patient selection factors, no such advantage was observed in patients receiving immunotherapy. These findings suggest that modern systemic therapy may effectively mitigate the risks associated with limited axillary surgery, offering novel insights for clinical decision-making.

## Data Availability

The original contributions presented in the study are included in the article/**Supplementary Material**, further inquiries can be directed to the corresponding author/s.
