# Supplementary figures and images for: Survival outcomes of axillary de-escalation following neoadjuvant chemo-immunotherapy in clinically node-positive triple-negative breast cancer: a national cancer database study
Source: Front Immunol. 2026 Jul 8;17:1892648. doi: 10.3389/fimmu.2026.1892648 (PMC13388904; doi:10.3389/fimmu.2026.1892648)

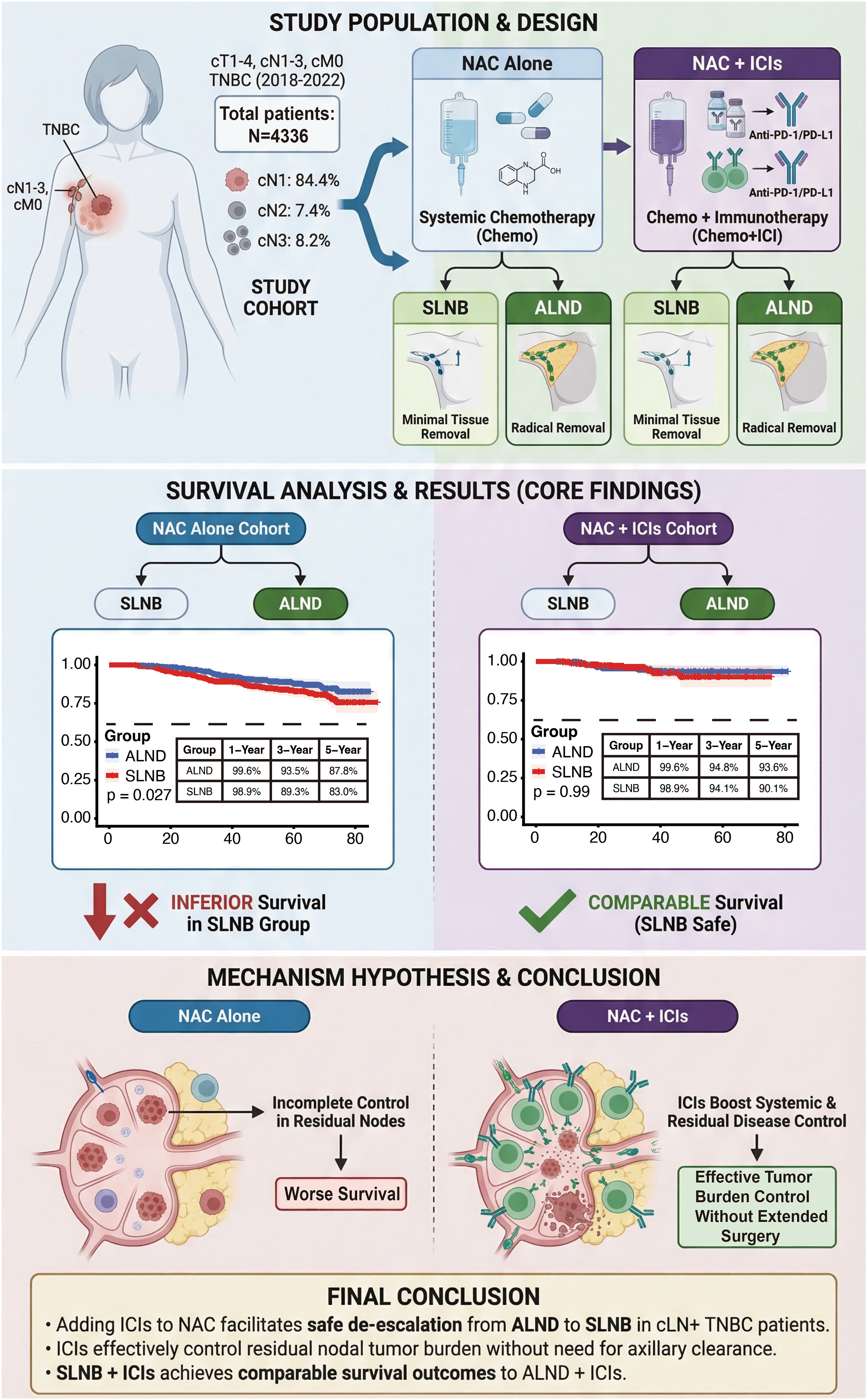

Supplement: Supplementary file 1 [file Image1.jpeg]
