# Supplementary material for: Survival outcomes of axillary de-escalation following neoadjuvant chemo-immunotherapy in clinically node-positive triple-negative breast cancer: a national cancer database study
Source: Front Immunol. 2026 Jul 8;17:1892648. doi: 10.3389/fimmu.2026.1892648 (PMC13388904; doi:10.3389/fimmu.2026.1892648)
Supplement: Supplementary file 2 [file Table1.docx]

**Supplementary Files:**

**Supplementary Figure 1. Evaluation of covariate balance and propensity score distribution before and after matching.**

(A) Distribution of propensity scores (dot plot) in the NAC cohort.

(B) Proportion of propensity score distribution in the NAC cohort.

(C) Distribution of propensity scores in the NAC+ICIs cohort.

(D) Proportion of propensity score distribution in the NAC+ICIs cohort.


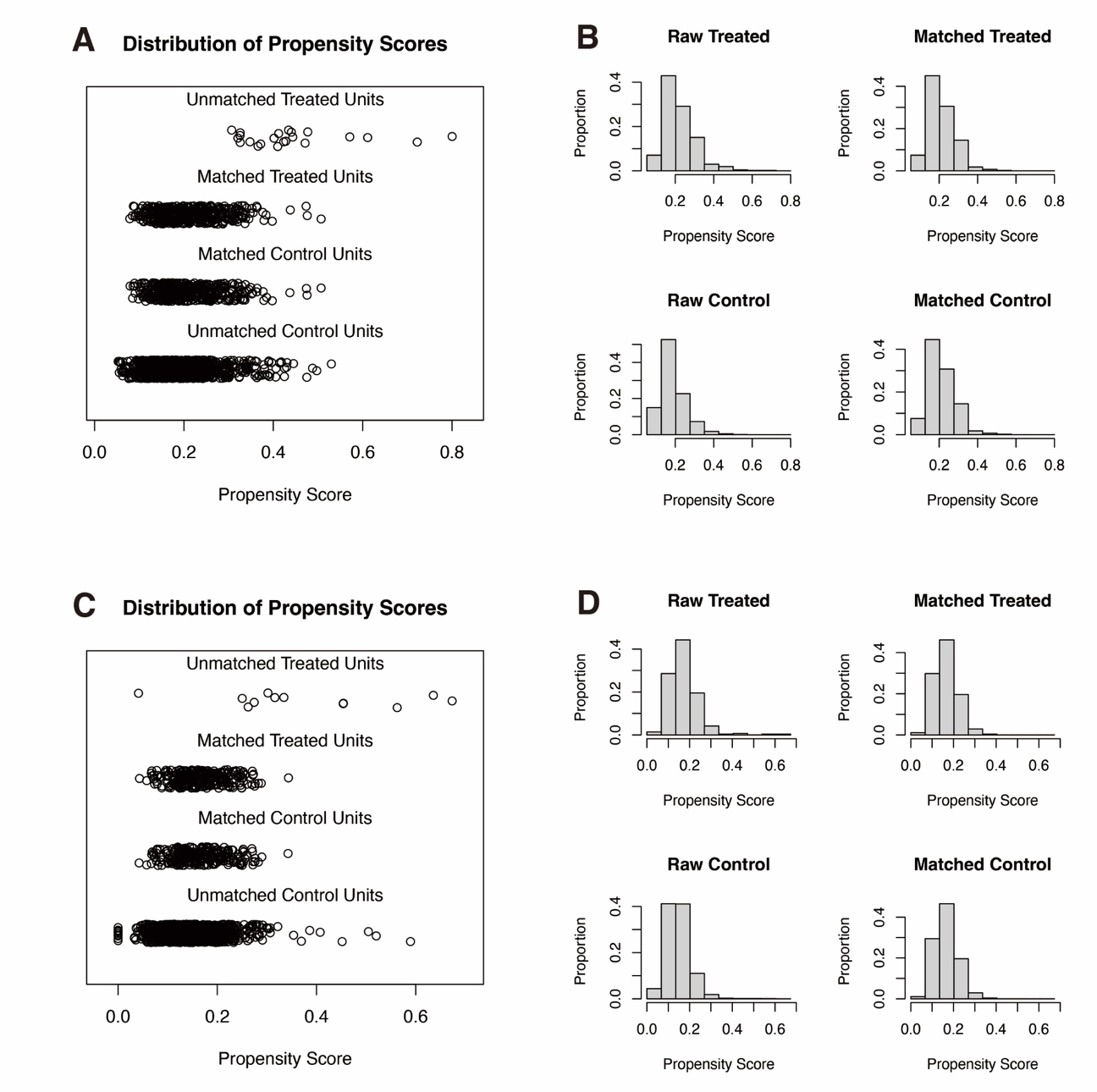


**Supplementary Figure 2. Survival analysis stratified by NAC and NAC+ICIs in (A) SLNB cohort and (B) ALND cohort.**


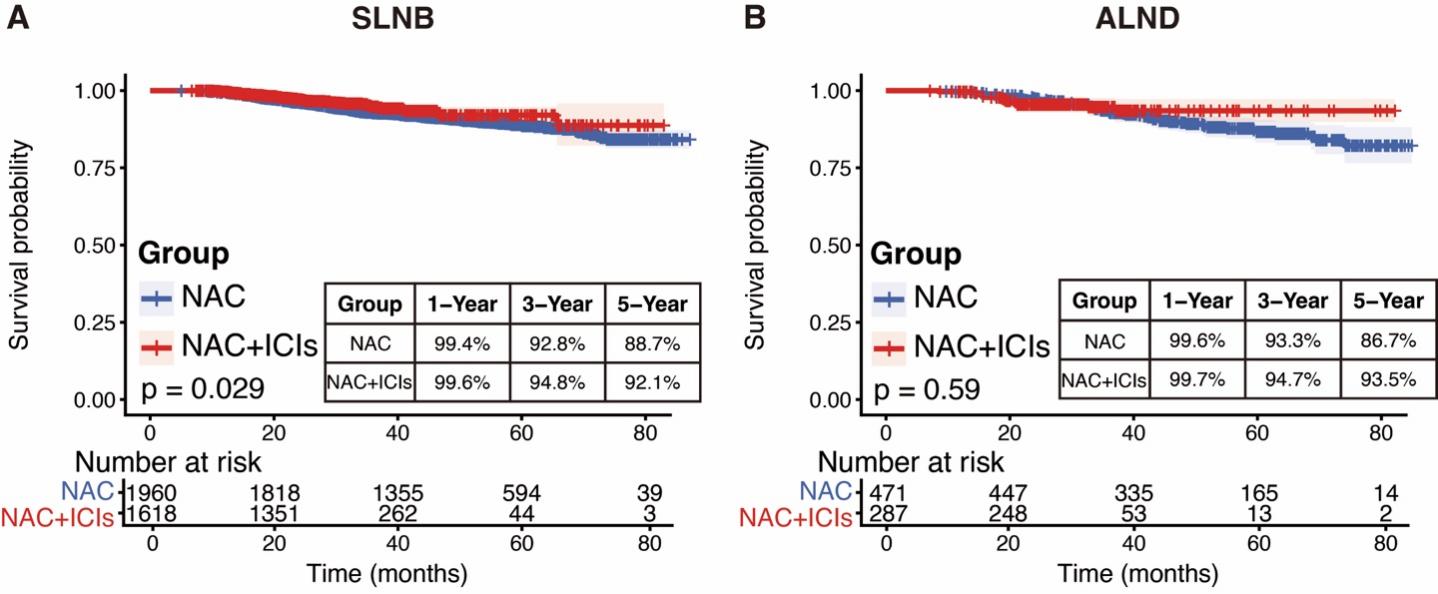


**Supplementary Figure 3. RMST analysis stratified by systemic therapy.**

(A) Patients in ALND cohort treated with NAC+ICIs

(B) Patients in ALND cohort treated with NAC

(C) Patients in SLNB cohort treated with NAC+ICIs

(D) Patients in SLNB cohort treated with NAC


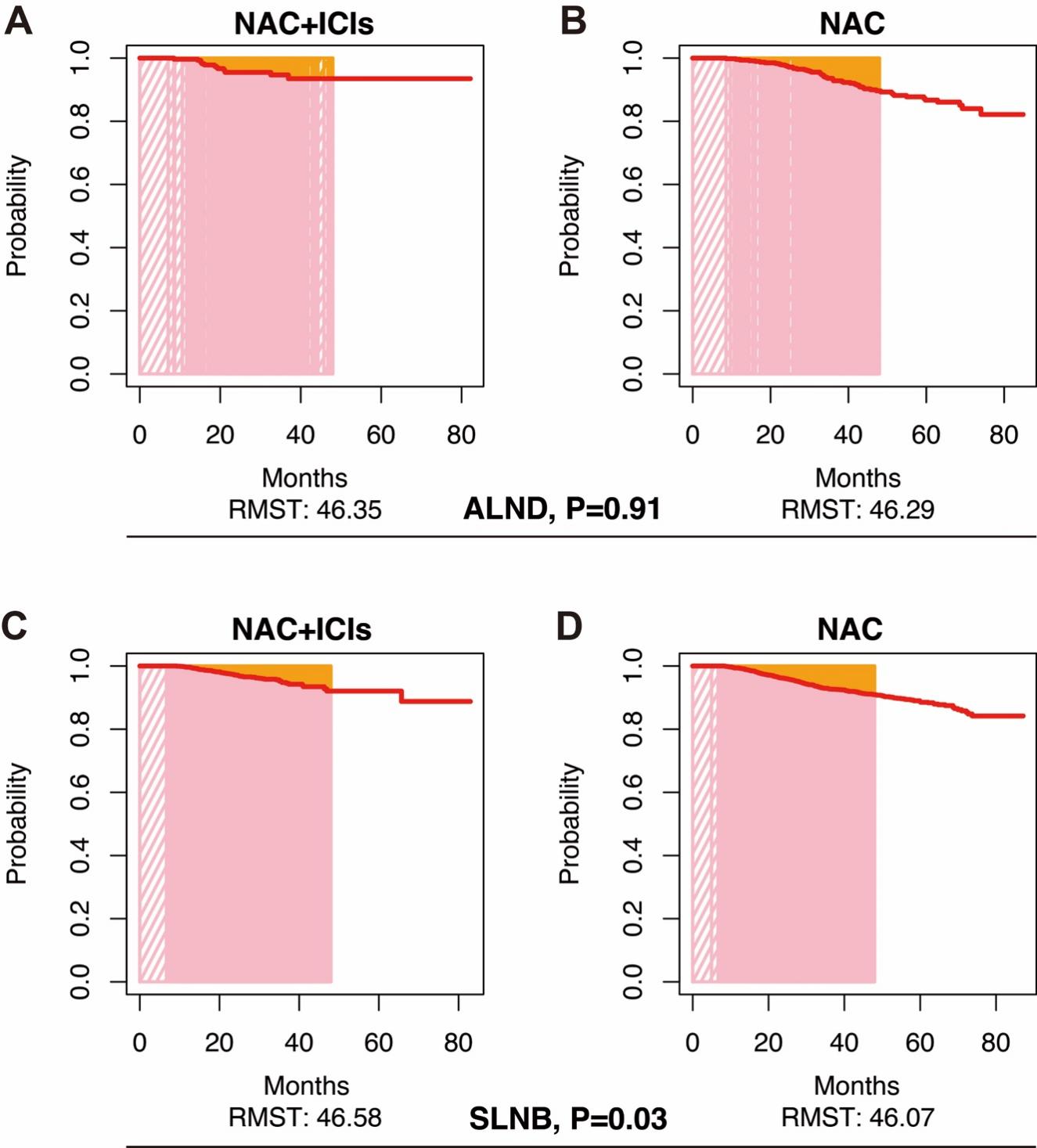


**Supplementary Table 1 Baseline characteristics of patients in matched NAC cohort**

|  | **Before PSM** | | |  | **After PSM** | | |
| --- | --- | --- | --- | --- | --- | --- | --- |
|  | **ALND** | **SLNB** | **P-value** |  | **ALND** | **SLNB** | **P-value** |
|  | **(N=471)** | **(N=1960)** |  |  | **(N=449)** | **(N=449)** |  |
| **Age** |  |  |  |  |  |  |  |
| **Mean (SD)** | **53.6 (13.0)** | **52.4 (13.0)** | **0.0697** |  | **53.4 (12.9)** | **53.8 (12.7)** | **0.619** |
| **Median [IQR]** | **53.0 [44.0, 63.0]** | **52.0 [43.0, 62.0]** |  |  | **53.0 [44.0, 63.0]** | **53.0 [45.0, 63.0]** |  |
| **Race (%)** |  |  |  |  |  |  |  |
| **White** | **298 (63.3%)** | **1292 (65.9%)** | **0.605** |  | **287 (63.9%)** | **282 (62.8%)** | **0.888** |
| **Asian and Pacific Islander** | **21 (4.5%)** | **88 (4.5%)** |  |  | **20 (4.5%)** | **15 (3.3%)** |  |
| **Black or African American** | **135 (28.7%)** | **531 (27.1%)** |  |  | **127 (28.3%)** | **137 (30.5%)** |  |
| **Unknown** | **15 (3.2%)** | **41 (2.1%)** |  |  | **13 (2.9%)** | **13 (2.9%)** |  |
| **American Indian or Alaska Native** | **2 (0.4%)** | **8 (0.4%)** |  |  | **2 (0.4%)** | **2 (0.4%)** |  |
| **Insurance (%)** |  |  |  |  |  |  |  |
| **Government** | **166 (35.2%)** | **700 (35.7%)** | **0.789** |  | **156 (34.7%)** | **164 (36.5%)** | **0.273** |
| **Not Insured** | **18 (3.8%)** | **64 (3.3%)** |  |  | **17 (3.8%)** | **15 (3.3%)** |  |
| **Private Insurance/Managed Care** | **281 (59.7%)** | **1179 (60.2%)** |  |  | **270 (60.1%)** | **269 (59.9%)** |  |
| **Unknown** | **6 (1.3%)** | **17 (0.9%)** |  |  | **6 (1.3%)** | **1 (0.2%)** |  |
| **Charlson-Deyo comorbidity index (%)** |  |  |  |  |  |  |  |
| **0** | **403 (85.6%)** | **1664 (84.9%)** | **0.603** |  | **385 (85.7%)** | **377 (84.0%)** | **0.385** |
| **1** | **47 (10.0%)** | **218 (11.1%)** |  |  | **45 (10.0%)** | **58 (12.9%)** |  |
| **2** | **12 (2.5%)** | **54 (2.8%)** |  |  | **12 (2.7%)** | **7 (1.6%)** |  |
| **≥3** | **9 (1.9%)** | **24 (1.2%)** |  |  | **7 (1.6%)** | **7 (1.6%)** |  |
| **Year of diagnosis (%)** |  |  |  |  |  |  |  |
| **2018** | **150 (31.8%)** | **407 (20.8%)** | **<0.001** |  | **133 (29.6%)** | **133 (29.6%)** | **0.729** |
| **2019** | **107 (22.7%)** | **576 (29.4%)** |  |  | **106 (23.6%)** | **115 (25.6%)** |  |
| **2020** | **111 (23.6%)** | **502 (25.6%)** |  |  | **110 (24.5%)** | **94 (20.9%)** |  |
| **2021** | **80 (17.0%)** | **370 (18.9%)** |  |  | **78 (17.4%)** | **86 (19.2%)** |  |
| **2022** | **23 (4.9%)** | **105 (5.4%)** |  |  | **22 (4.9%)** | **21 (4.7%)** |  |
| **Laterality (%)** |  |  |  |  |  |  |  |
| **left** | **235 (49.9%)** | **1042 (53.2%)** | **0.378** |  | **223 (49.7%)** | **261 (58.1%)** | **0.026** |
| **right** | **235 (49.9%)** | **916 (46.7%)** |  |  | **225 (50.1%)** | **188 (41.9%)** |  |
| **Unknown** | **1 (0.2%)** | **2 (0.1%)** |  |  | **1 (0.2%)** | **0 (0%)** |  |
| **Lymphovascular Invasion (%)** |  |  |  |  |  |  |  |
| **Yes** | **48 (10.2%)** | **194 (9.9%)** | **0.147** |  | **43 (9.6%)** | **47 (10.5%)** | **0.0953** |
| **No** | **263 (55.8%)** | **1006 (51.3%)** |  |  | **256 (57.0%)** | **224 (49.9%)** |  |
| **Unknown** | **160 (34.0%)** | **760 (38.8%)** |  |  | **150 (33.4%)** | **178 (39.6%)** |  |
| **Surgery type (%)** |  |  |  |  |  |  |  |
| **Breast-Conserving Surgery** | **214 (45.4%)** | **994 (50.7%)** | **0.0448** |  | **205 (45.7%)** | **230 (51.2%)** | **0.109** |
| **Total mastectomy** | **257 (54.6%)** | **966 (49.3%)** |  |  | **244 (54.3%)** | **219 (48.8%)** |  |
| **Surgical margins (%)** |  |  |  |  |  |  |  |
| **Negative** | **454 (96.4%)** | **1905 (97.2%)** | **0.438** |  | **432 (96.2%)** | **437 (97.3%)** | **0.575** |
| **Positive** | **10 (2.1%)** | **26 (1.3%)** |  |  | **10 (2.2%)** | **6 (1.3%)** |  |
| **Unknown** | **7 (1.5%)** | **29 (1.5%)** |  |  | **7 (1.6%)** | **6 (1.3%)** |  |
| **Postoperative radiation (%)** |  |  |  |  |  |  |  |
| **No** | **103 (21.9%)** | **365 (18.6%)** | **0.124** |  | **95 (21.2%)** | **89 (19.8%)** | **0.679** |
| **Yes** | **368 (78.1%)** | **1595 (81.4%)** |  |  | **354 (78.8%)** | **360 (80.2%)** |  |
| **Postoperative chemotherapy (%)** |  |  |  |  |  |  |  |
| **No** | **377 (80.0%)** | **1584 (80.8%)** | **0.751** |  | **363 (80.8%)** | **365 (81.3%)** | **0.932** |
| **Yes** | **94 (20.0%)** | **376 (19.2%)** |  |  | **86 (19.2%)** | **84 (18.7%)** |  |
| **cT stage (%)** |  |  |  |  |  |  |  |
| **cT1** | **114 (24.2%)** | **396 (20.2%)** | **0.0041** |  | **108 (24.1%)** | **108 (24.1%)** | **0.576** |
| **cT2** | **243 (51.6%)** | **1161 (59.2%)** |  |  | **239 (53.2%)** | **252 (56.1%)** |  |
| **cT3** | **82 (17.4%)** | **324 (16.5%)** |  |  | **80 (17.8%)** | **65 (14.5%)** |  |
| **cT4** | **32 (6.8%)** | **79 (4.0%)** |  |  | **22 (4.9%)** | **24 (5.3%)** |  |
| **cN stage (%)** |  |  |  |  |  |  |  |
| **cN1** | **390 (82.8%)** | **1684 (85.9%)** | **0.227** |  | **378 (84.2%)** | **380 (84.6%)** | **0.784** |
| **cN2** | **43 (9.1%)** | **144 (7.3%)** |  |  | **38 (8.5%)** | **33 (7.3%)** |  |
| **cN3** | **38 (8.1%)** | **132 (6.7%)** |  |  | **33 (7.3%)** | **36 (8.0%)** |  |
| **ypT stage (%)** |  |  |  |  |  |  |  |
| **ypT0/ypTis** | **302 (64.1%)** | **1305 (66.6%)** | **0.105** |  | **296 (65.9%)** | **311 (69.3%)** | **0.585** |
| **ypT1** | **127 (27.0%)** | **516 (26.3%)** |  |  | **118 (26.3%)** | **108 (24.1%)** |  |
| **ypT2** | **30 (6.4%)** | **119 (6.1%)** |  |  | **29 (6.5%)** | **23 (5.1%)** |  |
| **ypT3** | **10 (2.1%)** | **18 (0.9%)** |  |  | **5 (1.1%)** | **7 (1.6%)** |  |
| **ypT4** | **2 (0.4%)** | **2 (0.1%)** |  |  | **1 (0.2%)** | **0 (0%)** |  |
| **Grade (%)** |  |  |  |  |  |  |  |
| **Moderately differentiated** | **56 (11.9%)** | **214 (10.9%)** | **0.0265** |  | **51 (11.4%)** | **60 (13.4%)** | **0.335** |
| **Poorly differentiated** | **393 (83.4%)** | **1702 (86.8%)** |  |  | **382 (85.1%)** | **371 (82.6%)** |  |
| **Unknown** | **20 (4.2%)** | **41 (2.1%)** |  |  | **14 (3.1%)** | **18 (4.0%)** |  |
| **Well differentiated** | **2 (0.4%)** | **3 (0.2%)** |  |  | **2 (0.4%)** | **0 (0%)** |  |
| **pCR (%)** |  |  |  |  |  |  |  |
| **No** | **169 (35.9%)** | **655 (33.4%)** | **0.337** |  | **153 (34.1%)** | **138 (30.7%)** | **0.318** |
| **Yes** | **302 (64.1%)** | **1305 (66.6%)** |  |  | **296 (65.9%)** | **311 (69.3%)** |  |
| **Zip-code education (%)** |  |  |  |  |  |  |  |
| **< 5.0%** | **68 (14.4%)** | **355 (18.1%)** | **0.322** |  | **67 (14.9%)** | **60 (13.4%)** | **0.764** |
| **5.0% - 9.0%** | **115 (24.4%)** | **481 (24.5%)** |  |  | **109 (24.3%)** | **120 (26.7%)** |  |
| **9.1% - 15.2%** | **116 (24.6%)** | **454 (23.2%)** |  |  | **108 (24.1%)** | **101 (22.5%)** |  |
| **>15.3%** | **84 (17.8%)** | **352 (18.0%)** |  |  | **81 (18.0%)** | **90 (20.0%)** |  |
| **Unknown** | **88 (18.7%)** | **318 (16.2%)** |  |  | **84 (18.7%)** | **78 (17.4%)** |  |
| **Zip-code income (%)** |  |  |  |  |  |  |  |
| **< $46,277** | **75 (15.9%)** | **265 (13.5%)** | **0.201** |  | **71 (15.8%)** | **64 (14.3%)** | **0.503** |
| **$46,277 - $57,856** | **67 (14.2%)** | **348 (17.8%)** |  |  | **63 (14.0%)** | **82 (18.3%)** |  |
| **$57,857 - $74,062** | **99 (21.0%)** | **414 (21.1%)** |  |  | **96 (21.4%)** | **98 (21.8%)** |  |
| **>$74,063** | **141 (29.9%)** | **612 (31.2%)** |  |  | **134 (29.8%)** | **125 (27.8%)** |  |
| **Unknown** | **89 (18.9%)** | **321 (16.4%)** |  |  | **85 (18.9%)** | **80 (17.8%)** |  |
| **Urban/rural location (%)** |  |  |  |  |  |  |  |
| **Urban** | **36 (7.6%)** | **181 (9.2%)** | **0.129** |  | **35 (7.8%)** | **31 (6.9%)** | **0.927** |
| **Metro** | **415 (88.1%)** | **1651 (84.2%)** |  |  | **394 (87.8%)** | **395 (88.0%)** |  |
| **Rural** | **11 (2.3%)** | **56 (2.9%)** |  |  | **11 (2.4%)** | **13 (2.9%)** |  |
| **Unknown** | **9 (1.9%)** | **72 (3.7%)** |  |  | **9 (2.0%)** | **10 (2.2%)** |  |
| **Facility type (%)** |  |  |  |  |  |  |  |
| **Academic/Research Program** | **151 (32.1%)** | **568 (29.0%)** | **0.281** |  | **137 (30.5%)** | **158 (35.2%)** | **0.547** |
| **Community Cancer Program** | **25 (5.3%)** | **94 (4.8%)** |  |  | **25 (5.6%)** | **21 (4.7%)** |  |
| **Comprehensive Community Cancer Program** | **142 (30.1%)** | **555 (28.3%)** |  |  | **134 (29.8%)** | **134 (29.8%)** |  |
| **Integrated Network Cancer Program** | **83 (17.6%)** | **389 (19.8%)** |  |  | **83 (18.5%)** | **70 (15.6%)** |  |
| **Unknown** | **70 (14.9%)** | **354 (18.1%)** |  |  | **70 (15.6%)** | **66 (14.7%)** |  |

SLNB, sentinel lymph node biopsy. ALND, axillary lymph node dissection. cT stage, clinical T stage. cN stage, clinical N stage. ypT stage, post-therapy pathologic T stage; pCR, pathologic complete response

**Supplementary Table 2 Baseline characteristics of patients in matched NAC+ICIs cohort**

|  | **ALND** | **SLNB** | **P-value** |  | **ALND** | **SLNB** | **P-value** |
| --- | --- | --- | --- | --- | --- | --- | --- |
|  | **(N=287)** | **(N=1618)** |  |  | **(N=275)** | **(N=275)** |  |
| **Age** |  |  |  |  |  |  |  |
| **Mean (SD)** | **51.8 (13.6)** | **52.0 (12.9)** | **0.826** |  | **52.1 (13.7)** | **52.2 (12.8)** | **0.936** |
| **Median [IQR]** | **51.0 [41.0, 62.0]** | **52.0 [41.0, 62.0]** |  |  | **52.0 [42.0, 61.0]** | **52.0 [42.0, 61.0]** |  |
| **Race (%)** |  |  |  |  |  |  |  |
| **White** | **203 (70.7%)** | **1080 (66.7%)** | **0.00962** |  | **199 (72.4%)** | **191 (69.5%)** | **0.825** |
| **Asian and Pacific Islander** | **15 (5.2%)** | **96 (5.9%)** |  |  | **14 (5.1%)** | **15 (5.5%)** |  |
| **Black or African American** | **60 (20.9%)** | **391 (24.2%)** |  |  | **59 (21.5%)** | **64 (23.3%)** |  |
| **Unknown** | **4 (1.4%)** | **46 (2.8%)** |  |  | **3 (1.1%)** | **5 (1.8%)** |  |
| **American Indian or Alaska Native** | **5 (1.7%)** | **5 (0.3%)** |  |  | **NA** | **NA** |  |
| **Insurance (%)** |  |  |  |  |  |  |  |
| **Government** | **107 (37.3%)** | **532 (32.9%)** | **0.374** |  | **102 (37.1%)** | **88 (32.0%)** | **0.568** |
| **Not Insured** | **6 (2.1%)** | **32 (2.0%)** |  |  | **6 (2.2%)** | **5 (1.8%)** |  |
| **Private Insurance/Managed Care** | **173 (60.3%)** | **1038 (64.2%)** |  |  | **166 (60.4%)** | **180 (65.5%)** |  |
| **Unknown** | **1 (0.3%)** | **16 (1.0%)** |  |  | **1 (0.4%)** | **2 (0.7%)** |  |
| **Charlson-Deyo comorbidity index (%)** |  |  |  |  |  |  |  |
| **0** | **252 (87.8%)** | **1375 (85.0%)** | **0.397** |  | **241 (87.6%)** | **241 (87.6%)** | **0.988** |
| **1** | **28 (9.8%)** | **174 (10.8%)** |  |  | **27 (9.8%)** | **26 (9.5%)** |  |
| **2** | **4 (1.4%)** | **50 (3.1%)** |  |  | **4 (1.5%)** | **5 (1.8%)** |  |
| **≥3** | **3 (1.0%)** | **19 (1.2%)** |  |  | **3 (1.1%)** | **3 (1.1%)** |  |
| **Year of diagnosis (%)** |  |  |  |  |  |  |  |
| **2018** | **8 (2.8%)** | **26 (1.6%)** | **0.417** |  | **6 (2.2%)** | **7 (2.5%)** | **0.602** |
| **2019** | **12 (4.2%)** | **47 (2.9%)** |  |  | **10 (3.6%)** | **8 (2.9%)** |  |
| **2020** | **22 (7.7%)** | **108 (6.7%)** |  |  | **22 (8.0%)** | **33 (12.0%)** |  |
| **2021** | **82 (28.6%)** | **492 (30.4%)** |  |  | **80 (29.1%)** | **75 (27.3%)** |  |
| **2022** | **163 (56.8%)** | **945 (58.4%)** |  |  | **157 (57.1%)** | **152 (55.3%)** |  |
| **Laterality (%)** |  |  |  |  |  |  |  |
| **left** | **142 (49.5%)** | **799 (49.4%)** | **1** |  | **138 (50.2%)** | **151 (54.9%)** | **0.306** |
| **right** | **145 (50.5%)** | **819 (50.6%)** |  |  | **137 (49.8%)** | **124 (45.1%)** |  |
| **Lymphovascular Invasion (%)** |  |  |  |  |  |  |  |
| **Yes** | **27 (9.4%)** | **172 (10.6%)** |  |  | **26 (9.5%)** | **31 (11.3%)** | **0.342** |
| **No** | **166 (57.8%)** | **854 (52.8%)** | **0.285** |  | **158 (57.5%)** | **141 (51.3%)** |  |
| **Unknown** | **94 (32.8%)** | **592 (36.6%)** |  |  | **91 (33.1%)** | **103 (37.5%)** |  |
| **Surgery type (%)** |  |  |  |  |  |  |  |
| **Breast-Conserving Surgery** | **116 (40.4%)** | **836 (51.7%)** | **<0.001** |  | **110 (40.0%)** | **137 (49.8%)** | **0.0258** |
| **Total mastectomy** | **171 (59.6%)** | **782 (48.3%)** |  |  | **165 (60.0%)** | **138 (50.2%)** |  |
| **Surgical margins (%)** |  |  |  |  |  |  |  |
| **Negative** | **280 (97.6%)** | **1559 (96.4%)** | **0.571** |  | **268 (97.5%)** | **262 (95.3%)** | **0.194** |
| **Positive** | **2 (0.7%)** | **20 (1.2%)** |  |  | **2 (0.7%)** | **1 (0.4%)** |  |
| **Unknown** | **5 (1.7%)** | **39 (2.4%)** |  |  | **5 (1.8%)** | **12 (4.4%)** |  |
| **Postoperative radiation (%)** |  |  |  |  |  |  |  |
| **No** | **68 (23.7%)** | **277 (17.1%)** | **0.00983** |  | **64 (23.3%)** | **63 (22.9%)** | **1** |
| **Yes** | **219 (76.3%)** | **1341 (82.9%)** |  |  | **211 (76.7%)** | **212 (77.1%)** |  |
| **Postoperative chemotherapy (%)** |  |  |  |  |  |  |  |
| **No** | **153 (53.3%)** | **792 (48.9%)** | **0.194** |  | **147 (53.5%)** | **138 (50.2%)** | **0.495** |
| **Yes** | **134 (46.7%)** | **826 (51.1%)** |  |  | **128 (46.5%)** | **137 (49.8%)** |  |
| **cT stage (%)** |  |  |  |  |  |  |  |
| **cT1** | **50 (17.4%)** | **324 (20.0%)** | **0.149** |  | **50 (18.2%)** | **39 (14.2%)** | **0.483** |
| **cT2** | **158 (55.1%)** | **925 (57.2%)** |  |  | **151 (54.9%)** | **153 (55.6%)** |  |
| **cT3** | **61 (21.3%)** | **308 (19.0%)** |  |  | **58 (21.1%)** | **69 (25.1%)** |  |
| **cT4** | **18 (6.3%)** | **61 (3.8%)** |  |  | **16 (5.8%)** | **14 (5.1%)** |  |
| **cN stage (%)** |  |  |  |  |  |  |  |
| **cN1** | **239 (83.3%)** | **1345 (83.1%)** | **0.643** |  | **231 (84.0%)** | **234 (85.1%)** | **0.935** |
| **cN2** | **23 (8.0%)** | **111 (6.9%)** |  |  | **21 (7.6%)** | **20 (7.3%)** |  |
| **cN3** | **25 (8.7%)** | **162 (10.0%)** |  |  | **23 (8.4%)** | **21 (7.6%)** |  |
| **ypT stage (%)** |  |  |  |  |  |  |  |
| **ypT0/ypTis** | **229 (79.8%)** | **1276 (78.9%)** | **0.93** |  | **221 (80.4%)** | **219 (79.6%)** | **0.938** |
| **ypT1** | **46 (16.0%)** | **281 (17.4%)** |  |  | **43 (15.6%)** | **47 (17.1%)** |  |
| **ypT2** | **11 (3.8%)** | **53 (3.3%)** |  |  | **10 (3.6%)** | **8 (2.9%)** |  |
| **ypT3** | **1 (0.3%)** | **6 (0.4%)** |  |  | **1 (0.4%)** | **1 (0.4%)** |  |
| **ypT4** | **0 (0%)** | **2 (0.1%)** |  |  | **NA** | **NA** |  |
| **Grade (%)** |  |  |  |  |  |  |  |
| **Moderately differentiated** | **18 (6.3%)** | **167 (10.3%)** | **0.181** |  | **18 (6.5%)** | **20 (7.3%)** | **0.327** |
| **Poorly differentiated** | **262 (91.3%)** | **1411 (87.2%)** |  |  | **251 (91.3%)** | **243 (88.4%)** |  |
| **Unknown** | **7 (2.4%)** | **32 (2.0%)** |  |  | **6 (2.2%)** | **12 (4.4%)** |  |
| **Undifferentiated and anaplastic** | **0 (0%)** | **2 (0.1%)** |  |  | **NA** | **NA** |  |
| **Well differentiated** | **0 (0%)** | **6 (0.4%)** |  |  | **NA** | **NA** |  |
| **pCR (%)** |  |  |  |  |  |  |  |
| **No** | **58 (20.2%)** | **342 (21.1%)** | **0.782** |  | **54 (19.6%)** | **56 (20.4%)** | **0.915** |
| **Yes** | **229 (79.8%)** | **1276 (78.9%)** |  |  | **221 (80.4%)** | **219 (79.6%)** |  |
| **Zip-code education (%)** |  |  |  |  |  |  |  |
| **< 5.0%** | **50 (17.4%)** | **322 (19.9%)** | **0.44** |  | **48 (17.5%)** | **59 (21.5%)** | **0.246** |
| **5.0% - 9.0%** | **65 (22.6%)** | **381 (23.5%)** |  |  | **64 (23.3%)** | **45 (16.4%)** |  |
| **9.1% - 15.2%** | **63 (22.0%)** | **381 (23.5%)** |  |  | **60 (21.8%)** | **58 (21.1%)** |  |
| **>15.3%** | **46 (16.0%)** | **250 (15.5%)** |  |  | **44 (16.0%)** | **43 (15.6%)** |  |
| **Unknown** | **63 (22.0%)** | **284 (17.6%)** |  |  | **59 (21.5%)** | **70 (25.5%)** |  |
| **Zip-code income (%)** |  |  |  |  |  |  |  |
| **< $46,277** | **37 (12.9%)** | **162 (10.0%)** | **0.108** |  | **35 (12.7%)** | **21 (7.6%)** | **0.198** |
| **$46,277 - $57,856** | **52 (18.1%)** | **284 (17.6%)** |  |  | **48 (17.5%)** | **45 (16.4%)** |  |
| **$57,857 - $74,062** | **57 (19.9%)** | **350 (21.6%)** |  |  | **57 (20.7%)** | **50 (18.2%)** |  |
| **>$74,063** | **78 (27.2%)** | **537 (33.2%)** |  |  | **76 (27.6%)** | **89 (32.4%)** |  |
| **Unknown** | **63 (22.0%)** | **285 (17.6%)** |  |  | **59 (21.5%)** | **70 (25.5%)** |  |
| **Urban/rural location (%)** |  |  |  |  |  |  |  |
| **Urban** | **28 (9.8%)** | **120 (7.4%)** |  |  | **24 (8.7%)** | **26 (9.5%)** | **0.96** |
| **Metro** | **236 (82.2%)** | **1387 (85.7%)** | **0.459** |  | **229 (83.3%)** | **226 (82.2%)** |  |
| **Rural** | **7 (2.4%)** | **31 (1.9%)** |  |  | **7 (2.5%)** | **6 (2.2%)** |  |
| **Unknown** | **16 (5.6%)** | **80 (4.9%)** |  |  | **15 (5.5%)** | **17 (6.2%)** |  |
| **Facility type (%)** |  |  |  |  |  |  |  |
| **Academic/Research Program** | **90 (31.4%)** | **486 (30.0%)** | **0.703** |  | **85 (30.9%)** | **87 (31.6%)** | **0.992** |
| **Community Cancer Program** | **11 (3.8%)** | **69 (4.3%)** |  |  | **11 (4.0%)** | **11 (4.0%)** |  |
| **Comprehensive Community Cancer Program** | **77 (26.8%)** | **402 (24.8%)** |  |  | **74 (26.9%)** | **77 (28.0%)** |  |
| **Integrated Network Cancer Program** | **48 (16.7%)** | **327 (20.2%)** |  |  | **47 (17.1%)** | **43 (15.6%)** |  |
| **Unknown** | **61 (21.3%)** | **334 (20.6%)** |  |  | **58 (21.1%)** | **57 (20.7%)** |  |

SLNB, sentinel lymph node biopsy. ALND, axillary lymph node dissection. cT stage, clinical T stage. cN stage, clinical N stage. ypT stage, post-therapy pathologic T stage; pCR, pathologic complete response

**Supplementary Table 3 Detailed estimates from the RMST analysis stratified by SLNB and ALND.**

| **Analysis  (The truncation time: 48 months)** | **SLNB** | | |  | **ALND** | | |
| --- | --- | --- | --- | --- | --- | --- | --- |
|  | NAC+ICIs | **NAC** | **P-value** |  | NAC+ICIs | **NAC** | **P-value** |
|  | **Months (95%CI)** | **Months (95%CI)** |  |  | **Months (95%CI)** | **Months (95%CI)** |  |
| **Restricted mean survival time** | **46.580 (46.240 - 46.921)** | **46.072 (45.759 - 46.384)** | **0.031** |  | **46.350 (45.506 - 47.193)** | **46.289 (45.733 - 46.845)** | **0.906** |
| **Restricted mean time lost** | **1.420 (1.079 - 1.760)** | **1.928 (1.616 - 2.241)** | **0.038** |  | **1.650 (0.807 - 2.494)** | **1.711 (1.155 - 2.267)** | **0.907** |

SLNB, sentinel lymph node biopsy. ALND, axillary lymph node dissection. NAC, neoadjuvant chemotherapy; ICIs, immune checkpoint inhibitors. Cl, confidence interval.

**Supplementary Table 4 Detailed estimates from the RMST analysis of patients treated with NAC+ICIs.**

| **Analysis  (The truncation time: 48 months)** | **NAC+ICIs** | | |
| --- | --- | --- | --- |
|  | **SLNB** | **ALND** | **P-value** |
|  | **Months(95%CI)** | **Months(95%CI)** |  |
| **Restricted mean survival time** | **46.386 (45.517 - 47.254)** | **46.386 (45.529 - 47.243)** | **0.999** |
| **Restricted mean time lost** | **1.614 (0.746 - 2.483)** | **1.614 (0.757 - 2.471)** | **0.999** |

SLNB, sentinel lymph node biopsy. ALND, axillary lymph node dissection. NAC, neoadjuvant chemotherapy; ICIs, immune checkpoint inhibitors. Cl, confidence interval.
